# Inducible high-efficiency CRISPR-Cas9-targeted gene editing and precision base editing in African trypanosomes

**DOI:** 10.1038/s41598-018-26303-w

**Published:** 2018-05-21

**Authors:** Eva Rico, Laura Jeacock, Julie Kovářová, David Horn

**Affiliations:** Wellcome Trust Centre for Anti-Infectives Research, School of Life Sciences, University of Dundee, Dow Street, Dundee, DD1 5EH UK

## Abstract

The Cas9 endonuclease can be programmed by guide RNA to introduce sequence-specific breaks in genomic DNA. Thus, Cas9-based approaches present a range of novel options for genome manipulation and precision editing. African trypanosomes are parasites that cause lethal human and animal diseases. They also serve as models for studies on eukaryotic biology, including ‘divergent’ biology. Genome modification, exploiting the native homologous recombination machinery, has been important for studies on trypanosomes but often requires multiple rounds of transfection using selectable markers that integrate at low efficiency. We report a system for delivering tetracycline inducible Cas9 and guide RNA to *Trypanosoma brucei*. In these cells, targeted DNA cleavage and gene disruption can be achieved at close to 100% efficiency without further selection. Disruption of aquaglyceroporin (*AQP2*) or amino acid transporter genes confers resistance to the clinical drugs pentamidine or eflornithine, respectively, providing simple and robust assays for editing efficiency. We also use the new system for homology-directed, precision base editing; a single-stranded oligodeoxyribonucleotide repair template was delivered to introduce a single *AQP2 -* T^791^G/L^264^R mutation in this case. The technology we describe now enables a range of novel programmed genome-editing approaches in *T*. *brucei* that would benefit from temporal control, high-efficiency and precision.

## Introduction

The African trypanosomes of the *Trypanosoma brucei* group are transmitted by tsetse flies and cause lethal diseases in humans and livestock, known as sleeping sickness and nagana, respectively. Research on these protozoan parasites, and related parasitic trypanosomatids, often focuses on virulence mechanisms and druggable biology^1^. However, their divergent position within the eukaryotic phylogeny, and the experimental tractability of *T*. *brucei* in particular, means they are also of interest as model Excavates for studies on various aspects of eukaryotic biology, including novel biology not seen in other eukaryotic model systems. Unusual features include an almost complete lack of introns, widespread polycistronic transcription and *trans*-splicing of every mRNA^2^.

The ability to manipulate parasite genomes has had a huge impact on this field of research^3^. In *T*. *brucei*, DNA constructs, typically introduced by electroporation, can be used for gene knockout, gene knockdown exploiting the native RNA interference (RNAi) machinery, tagging native alleles or inducible expression of recombinant products; robust inducible expression^4^ has been of particular value. Sequences towards the ends of the linearised DNA constructs serve as templates for homologous recombination reactions. Thus, targeted integration depends upon the native homologous recombination machinery; microhomology-mediated end-joining (MMEJ)^5^ and single-strand annealing^6^ also operate in *T*. *brucei*, while non-homologous end-joining appears to be absent. Targeted integration occurs at low frequency, however (typically <1 × 10^−5^ electroporated cells), meaning that antibiotic selectable marker genes must be integrated at a modified site followed by selection for the desired recombinant cells. Since the *T*. *brucei* core genome is diploid^7^, gene disruption approaches typically require two rounds of electroporation and selection.

Chromosomal site-specific DNA double strand breaks (DSBs) can increase recombination efficiency in *T*. *brucei*, as demonstrated following inducible expression of the I-*Sce*I meganuclease^8^. The same endonuclease can efficiently trigger inter-chromosomal homologous recombination^5^ or antigenic variation when breaks are targeted close to the expressed Variant Surface Glycoprotein gene^9,10^. For this approach, however, a specific 18-bp I-*Sce*I cleavage site must first be engineered at the desired target site, which limits its versatility. Cas9 is an RNA-guided DNA endonuclease that can be programmed in a sequence-specific manner using a short (17–20 b) synthetic guide RNA sequence; specific targeting involves RNA:DNA base-pairing. The only additional requirement is that this ‘protospacer’ sequence must immediately precede a protospacer adjacent motif (PAM; at the DNA target site but not to be included in the gRNA). The PAM recognized by the most widely used Cas9 is 5′-NGG, for the type II system from *Streptococcus pyogens*^11^. It is this tremendous versatility that has made Cas9 repurposing so popular for genome manipulation and editing^12^. Cas9 systems function naturally as bacterial adaptive immune systems. In the bacterial cells, Clustered Regularly Interspaced Short Palindromic Repeats (CRISPR), derived from invading phage DNA, produce CRISPR RNA (crRNA) which, together with a *trans*-activating crRNA (tracrRNA) program Cas9 (CRISPR associated protein 9) to cut both the RNA-complementary and non-complementary strands, producing DSBs in the target DNA, 3 bp upstream of the PAM. The crRNA and tracrRNA can be fused to generate a chimeric single guide RNA (sgRNA)^11^, such that a two-component system requires only Cas9 and an sgRNA for programmed editing in a heterologous system.

The application of Cas9-based genome editing to trypanosomatids has progressed rapidly in the last few years. For example, *Sp*Cas9 has been used to edit genes in *T*. *brucei* with a cloning-free approach, allowing for one-step gene knockout, but still requiring integration of antibiotic selectable markers at the target sites^13^. Another recent report describes the delivery of *in vitro* assembled ribonucleoproteins containing the smaller *Staphylococcus aureus* Cas9 for editing in trypanosomatid parasites; *T*. *brucei*, *Trypanosoma cruzi* and *Leishmania major*. In this case, a GFP-reporter was disrupted in >50% of targeted *T*. *brucei* cells^14^; the PAM recognized by *Sa*Cas9 is 5′-NNGRRT, where R represents A or G. *Sp*Cas9 has also been used to edit genes in the other trypanosomatids^15^, including those for which loss-of-function studies have previously been hampered, notably due to the absence of a native RNAi machinery^16^. Thus, these advances will have a major impact on tractability in terms of genetic manipulation in *T*. *cruzi*^17–19^, and *Leishmania spp*^13,20–23^.

Cas9-based approaches will also enable a range of new approaches in *T*. *brucei*. To date, however, an inducible Cas9 editing system has not yet been reported for any trypanosomatid. In *T*. *brucei*, double-allele editing without selectable markers has not yet been reported, double-allele editing efficiencies have not been determined and precision base editing is not readily achievable. We sought to address three specific issues to advance Cas9-based editing tools and approaches for *T*. *brucei*. Since current systems in protozoan parasites often use constitutive Cas9 expression, which can be toxic and continually risks off-target editing^18,24^, we first wanted to establish an inducible system. Second, we wanted to develop a system that allowed for DSB formation (and repair) in close to 100% of cells in the population in a week or less. Third, we wanted to use the system for template-directed precision base editing. By manipulating genes associated with resistance to clinical drugs, we report the successful attainment of all three goals.

## Results

### A system for inducible Cas9 expression and sgRNA expression in *T*. *brucei*

**Figure 1:**
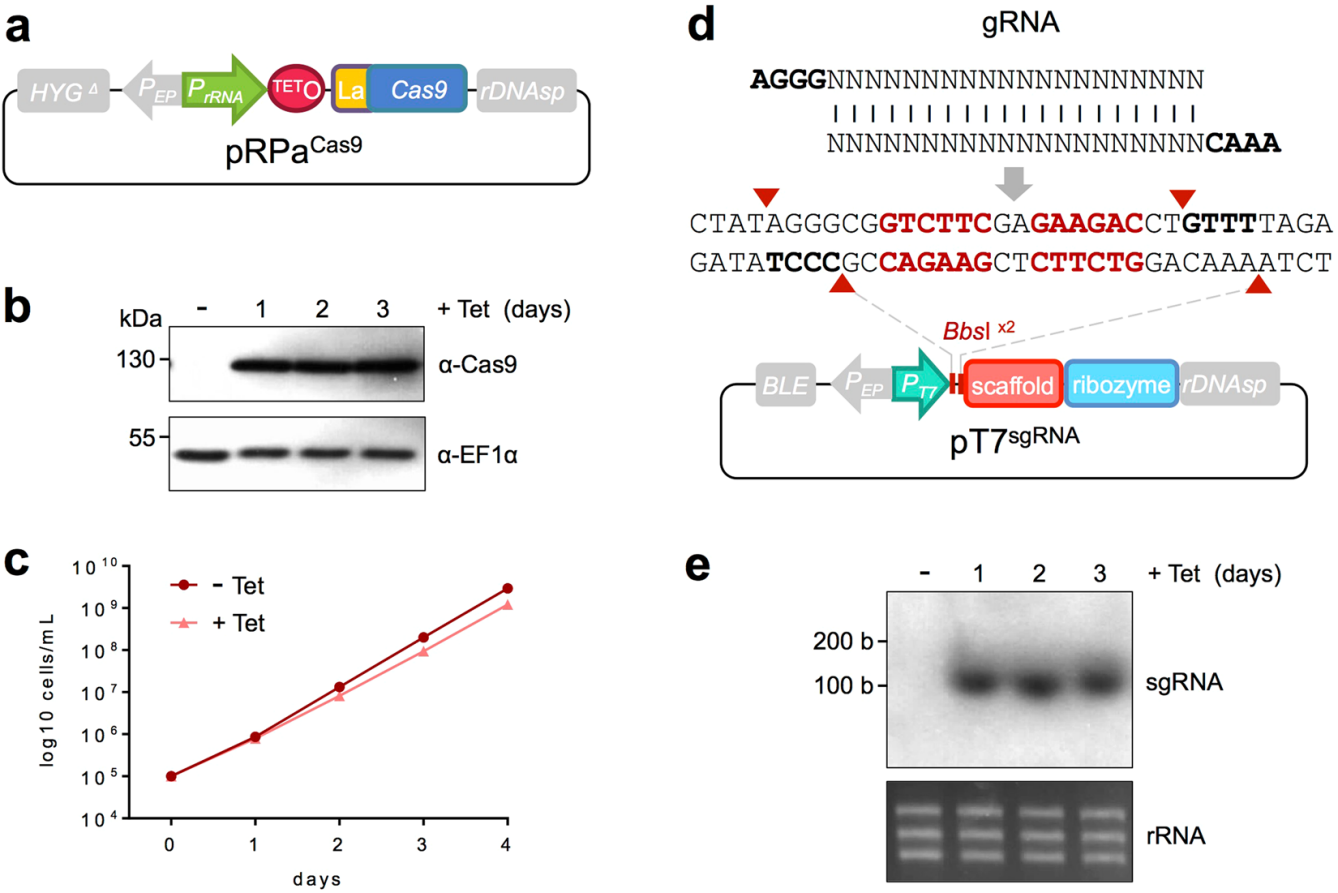
An inducible Cas9 system. (**a**) Schematic map of the pRPa^Cas9^ construct. *P*_*rRNA*_, *rRNA* promoter; TET_O_, tetracycline operator; La, nuclear localisation signal. Other components are from the pRPa parent vector (see the text for more details). (**b**) The protein blots show tightly regulated and inducible Cas9 expression (predicted mass: 159.4 kDa). The EF1α panel provides a loading control. Multiple independent clones yielded similar results. (**c**) Cell growth with (+Tet) and without (−Tet) Cas9 induction. Error bars (SD) from triplicate counts are obscured by the data symbols. A second independent clone gave similar results. (**d**) Schematic map of the pT7^sgRNA^ construct showing the gRNA cloning strategy using tandem *Bbs*I sites. The sgRNA scaffold and HDV ribozyme are also shown. *P*_*T7*_, phage T7 promoter. Other components are from the pRP^c6MYCn^ parent vector. (**e**) The RNA blot shows expression of the predicted 96 b sgRNA^AQP2^ that is dependent upon Cas9 expression. The rRNA panel provides a loading control. A second independent clone yielded similar results.

We assembled an expression construct with a tetracycline (Tet)-inducible ribosomal RNA (*rRNA*) promoter to drive *Sp*Cas9 expression in *T*. *brucei*. These pRPa constructs can be used in combination with *T*. *brucei* bloodstream-form 2T1 strains^25^, which express the Tet-repressor and have a partial hygromycin (*HYG*) drug-selectable marker gene at a non-transcribed *rDNA* spacer locus on chromosome 2; the expression construct contains a complementing portion of the *HYG* gene^26^. This allows for selection of hygromycin-resistant clones with expression cassettes reproducibly integrated at the same locus, a locus that supports robust and uniform Tet-inducible expression^26^. A synthetic *Sp*Cas9 gene with a La nuclear localisation signal^27^ at the *N*-terminus was codon-optimised for expression in *T*. *brucei*^28^. This gene was cloned in the pRPa expression construct and the resulting pRPa^Cas9^ plasmid (Fig. 1a) was linearised and introduced into 2T1^T7^ cells; these cells also express the phage T7 polymerase to drive sgRNA expression (see below). Analysis of the resulting 2T1^T7-Cas9^ clones revealed tightly regulated and robust Cas9 expression (Fig. 1b, Supplementary Fig. 1). We observed a moderate reduction in growth rate associated with Cas9 induction (Fig. 1c), which could reflect off-target editing in the absence of sgRNA^24^.

For expression of the sgRNA, a phage T7 promoter, a cloning site, the sgRNA scaffold and a hepatitis delta virus (HDV) ribozyme^29^ were synthesised in tandem (Fig. 1d). The cloning site comprises dual *Bbs*I restriction enzyme cleavage sites, which allows for high-efficiency cloning of annealed oligonucleotide pairs (AGGG[N]_20_/AAAC[N]_20_, Fig. 1d) without introducing additional unnecessary flanking sequences. The HDV ribozyme is a self-cleaving RNA that liberates the upstream RNA. Thus, the ribozyme generates the 3′-end of the sgRNA. This synthetic cassette was used to generate pT7^sgRNA^ that integrates at a second non-transcribed *rDNA* spacer locus (Fig. 1d); there are estimated to be 10–20 *rDNA* spacer loci in the *T*. *brucei* genome^7^.

We selected the aquaglyceroporin gene, *AQP2*, (Tb927.10.14170) as a suitable target for editing since this gene is involved in drug resistance and parasites are resistant to pentamidine when *AQP2* is defective^30^. The full complement of >9,000 *T*. *brucei* protein-coding genes comprises 51% G + C content^7^ so suitable PAMs can be found approximately once every 8 bp in these sequences. We identified potential PAMs in *AQP2*, selected a suitable 20 b *AQP2*-specific gRNA sequence, annealed the cognate oligonucleotide pair and ligated the product to *Bbs*I-digested pT7^sgRNA^. The resulting construct was linearised and introduced into 2T1^T7-Cas9^ cells. We analysed two independent Cas9/sgRNA^AQP2^ clones and observed robust and reproducible expression of sgRNA of the expected size (96 b), which was dependent upon Cas9 expression (Fig. 1e, Supplementary Fig. 1). This suggests that sgRNA is unstable in the absence of Cas9. Thus, sgRNA is efficiently processed by the HDV ribozyme and is specifically stabilised by Cas9, providing evidence for physical interaction of these two components and assembly of Cas9 ribonucleoproteins in *T*. *brucei*.

### Tightly regulated and high-efficiency *AQP2* editing

**Figure 2:**
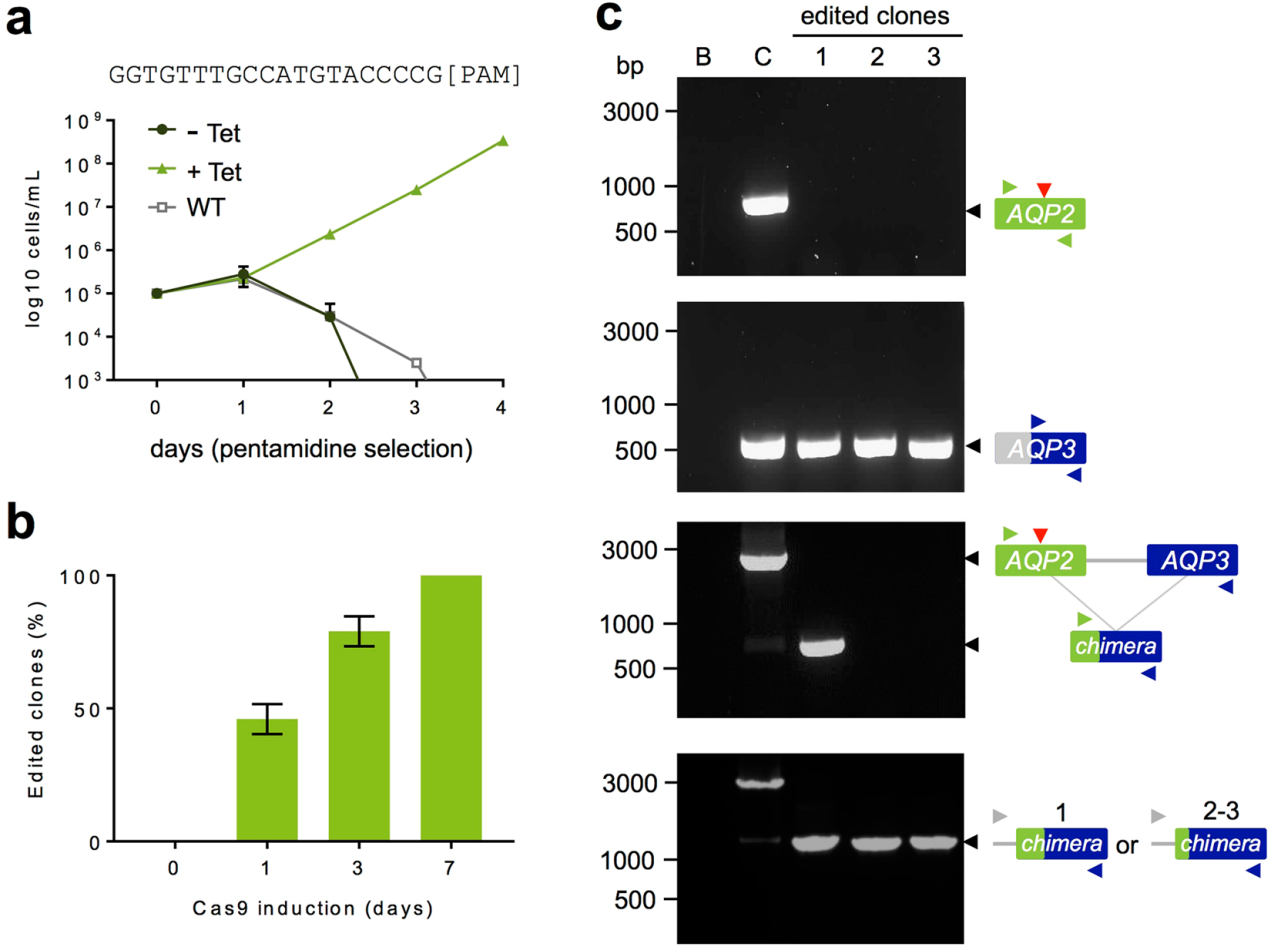
High-efficiency Cas9-based editing of the *AQP2* gene. (**a**) Cumulative cell growth in the presence of 6 nM pentamidine (estimated 2 × EC_50_) following 3-days with (+Tet) or without (−Tet) Cas9 induced editing of *AQP2*. Three independent Cas9/sgRNA^AQP2^ clones and wild-type (WT) cells were analysed. Error bars, SD. The gRNA sequence is shown above the plot. (**b**) A cloning assay reveals efficient Cas9 editing. Cas9 was induced as indicated followed by sub-cloning in the absence of Tet. The sub-clones were then transferred to medium with or without 6 nM pentamidine and considered ‘edited’ if drug-resistant; twelve sub-clones were analysed from each of two independent Cas9/sgRNA^AQP2^ clones (n = 24 sub-clones) for each time-point. (**c**) The PCR assays reveal *AQP2* editing in three independent drug-resistant clones. B, blank – no genomic DNA; C, control – wild-type genomic DNA. The *AQP3* gene is adjacent to *AQP2*. The vertical (red) arrowheads indicate the Cas9 target site in *AQP2*. The horizontal (green, blue and grey) arrowheads indicate the primers used for the PCR-assays. Predicted wild-type *AQP2* fragment, 724 bp; predicted *AQP3* fragment, 508 bp; predicted wild-type *AQP2-3* fragments in bottom two panels, 2,440 and 3,200 bp, respectively.

The *T*. *brucei* genome is diploid, meaning that Cas9-based editing for the vast majority of genes will involve targeting two identical sequences, ideally simultaneously in many cases. When AQP2 function is lost, the effective concentration of pentamidine to reduce growth by 50%, or the EC_50_, is approximately 15-fold greater than for wild-type cells^30^. Thus, pentamidine can be used to provide a robust phenotypic read-out and for quantitative analysis of bi-allelic editing. Using three independent Cas9/sgRNA^AQP2^ clones, we pre-induced Cas9 programmed to target *AQP2* for three days, and then selected with pentamidine. The growth profiles indicated an absence of drug-resistance without induction and robust drug-resistance with induction (Fig. 2a).

Encouraged by these results, using two independent Cas9/sgRNA^AQP2^ clones, we induced editing in the absence of pentamidine selection and sub-cloned the population either prior to induction, or one, three and seven days later. Under these conditions, un-edited, drug-sensitive cells remain viable and edited cells have no selective advantage. Twelve sub-clones from each clone and at each time-point were expanded under conditions where Cas9 was no longer induced and then tested for pentamidine resistance. This revealed remarkably tightly regulated editing (Fig. 2b). The absence of pentamidine-resistant sub-clones (0/24) from the uninduced population provided strong evidence for efficient temporal control, and this ‘baseline’ data allowed us to accurately assess editing during the induction time-course. This revealed efficiently induced editing, which increased from ~45% resistant clones after 1-day to ~80% after 3-days and reached the maximum 100% (24/24 sub-clones) after 7-days (Fig. 2b). This temporal response reflects the kinetics of Cas9 induction^4^, the Cas9 target search^31^ and DNA repair in *T*. *brucei*^32^, and may also be impacted by multiple rounds of single-allele break and error-free repair by homologous recombination. A series of PCR-based assays were used to assess the *AQP2-3* locus in three independent resistant clones (Fig. 2c, Supplementary Fig. 1). *AQP2* had indeed been disrupted (Fig. 2c, top panel) and replaced by *AQP2-3* chimeras in all three clones (Fig. 2c, lower panels), similar to what has been seen previously in drug-resistant isolates from relapsed patients^33,34^. Cloning and sequencing of the products revealed recombination within a block of identity from 168–222 nt in *AQP2* in clone 1, meaning that from 223 nt into the CDS is *AQP3* sequence in the chimera, and recombination within a block of identity from 1–91 nt in *AQP2* in clones 2–3, meaning that from 92 nt into the CDS is *AQP3* sequence in these chimeras. The chimeras likely arise by single-strand annealing, whereby single-stranded regions extend to adjacent repeated sequences and complementary strands anneal, leading to repair and the loss of one repeat^35^; the PCR-assays indicate loss of ~1.8 kbp in each case (Fig. 2c, lower panels), which is the size of the semi-repetitive *AQP2-3* region. Microhomology-mediated end-joining is more likely to be involved in repair at loci that lack extensive blocks of adjacent homology^5^. Thus, our inducible Cas9 editing system is tightly regulated and achieves temporally controlled and remarkably efficient bi-allelic editing.

### Tightly regulated and high-efficiency *AAT6* editing

**Figure 3:**
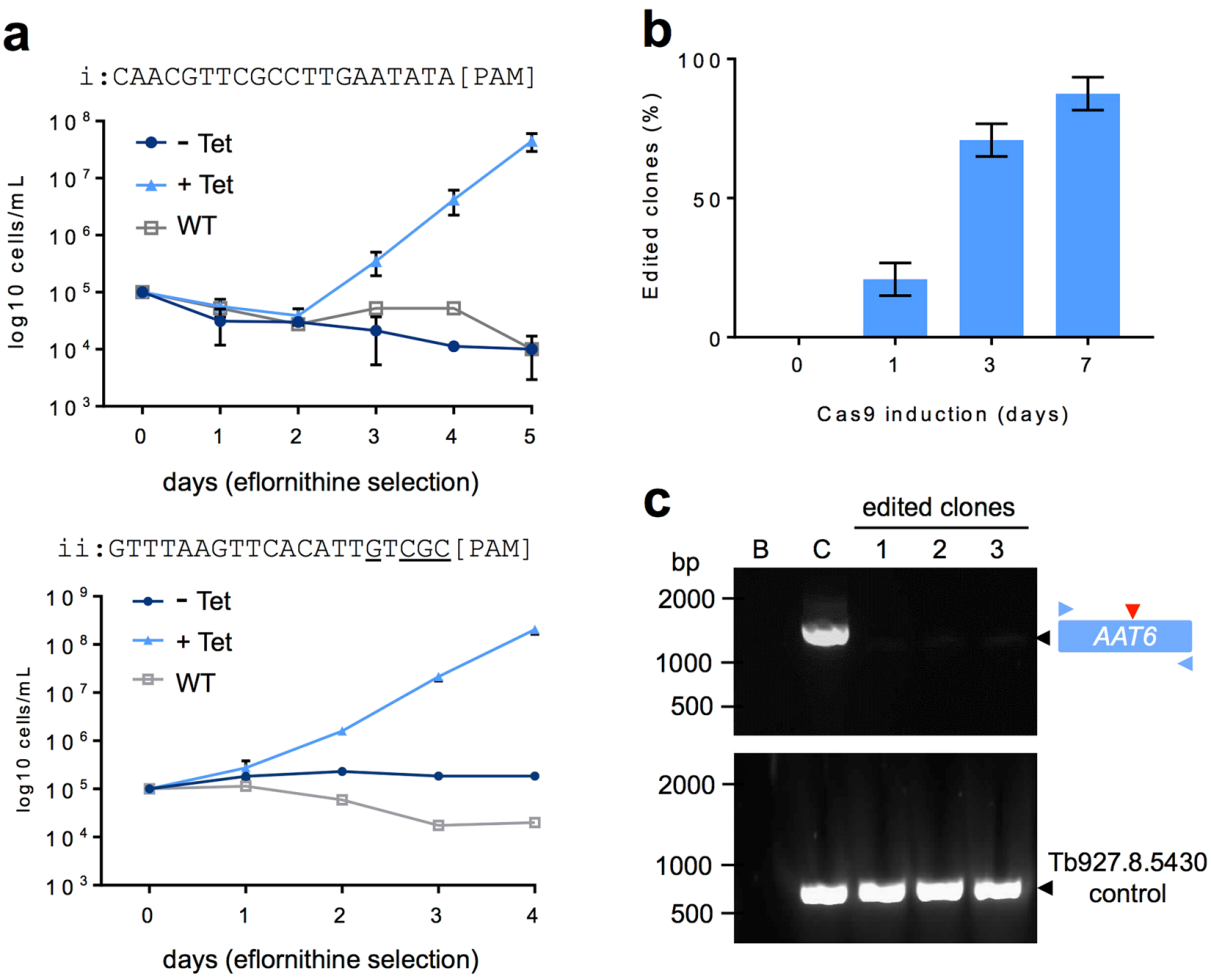
High efficiency Cas9-based editing of the *AAT6* gene. (**a**) Cumulative cell growth in the presence of 270 µM eflornithine (estimated 10 × EC_50_) following 3-days with (+Tet) or without (−Tet) Cas9 induced editing of *AAT6*. Two independent Cas9/sgRNA^AAT6^ clones and wild-type (WT) cells were analysed for each gRNA (i and ii). Error bars, SD. The gRNA sequences are shown above the plot with PAM-adjacent G/C residues underlined (see text for details). As can be seen, eflornithine is a cytostatic rather than cytocidal drug. (**b**) A cloning assay reveals efficient Cas9 editing by gRNA-ii. Cas9 was induced as indicated followed by sub-cloning in the absence of Tet. The sub-clones were then transferred to medium with or without 270 µM eflornithine and considered ‘edited’ if drug-resistant. Twelve sub-clones were analysed from each of two independent clones (n = 24 sub-clones) for each time-point. (**c**) The PCR assays reveal *AAT6* editing in three independent drug-resistant clones. B, blank – no genomic DNA; C, control – wild-type genomic DNA. The Tb927.8.5430 gene is close to *AAT6* and serves as an unedited control. The vertical (red) arrowhead indicates the Cas9 target site. The horizontal (blue) arrowheads indicate the primers used for the *AAT6* PCR-assay. Predicted wild-type *AAT6* fragment, 1,467 bp; predicted wild-type Tb927.8.5430 fragment, 648 bp.

To determine whether the efficiency of our system could be extended to another native locus, and to further test sgRNA design for *T*. *brucei*, we selected a second gene, on a different chromosome, for analysis. Again exploiting a known drug-resistance mechanism, we selected the amino acid transporter, or *AAT6* gene (Tb927.8.5450). Trypanosomes become resistant to the amino acid analogue, eflornithine when *AAT6* is defective and the eflornithine EC_50_ is >40-fold greater than for wild-type cells in this case^36^. Thus, eflornithine can also be used to provide a robust phenotypic read-out for bi-allelic editing. Since a PAM-proximal GC-content of >50% (at least 4 of 6 bases) yields optimal efficiency gRNAs in other cell-types^37^, we cloned two 20 b *AAT6*-specific gRNA sequences in pT7^sgRNA^. One gRNA (i) has a PAM-adjacent GC content of 0/6 and the other (ii) has a PAM-adjacent GC content of 4/6; both had an overall GC-content of 40% (Fig. 3a). The resulting constructs were introduced into 2T1^T7-Cas9^ cells as above. Using two independent Cas9/sgRNA^AAT6^ clones for each sgRNA, we induced Cas9 programmed to target *AAT6* for three days, followed by selection with eflornithine. In both cases, the growth profiles indicated an absence of resistance without induction and robust resistance with induction (Fig. 3a). We observed a striking difference between sgRNA-i and sgRNA-ii, however. As predicted by previous studies^37^, the sgRNA with the optimised PAM-proximal GC-content was associated with more efficient editing; with sgRNA-ii yielding drug-resistant cells more rapidly than sgRNA-i during selection (Fig. 3a).

As above, and using the optimised sgRNA (AAT6-ii) we next induced editing in the absence of eflornithine and sub-cloned the populations. The proportions of eflornithine-resistant sub-clones revealed remarkably efficient editing, reaching ~90% after 7-days (Fig. 3b). PCR-based assays confirmed disruption of the *AAT6* gene in three independent resistant clones (Fig. 3c, Supplementary Fig. 1). The process, from gRNA design to validation of edited clones can be performed in approximately three weeks. Thus, our tightly regulated inducible Cas9 editing system achieves rapid, high-efficiency, bi-allelic editing at native chromosomal loci.

### Precise and ‘scar-less’ single base editing

**Figure 4:**
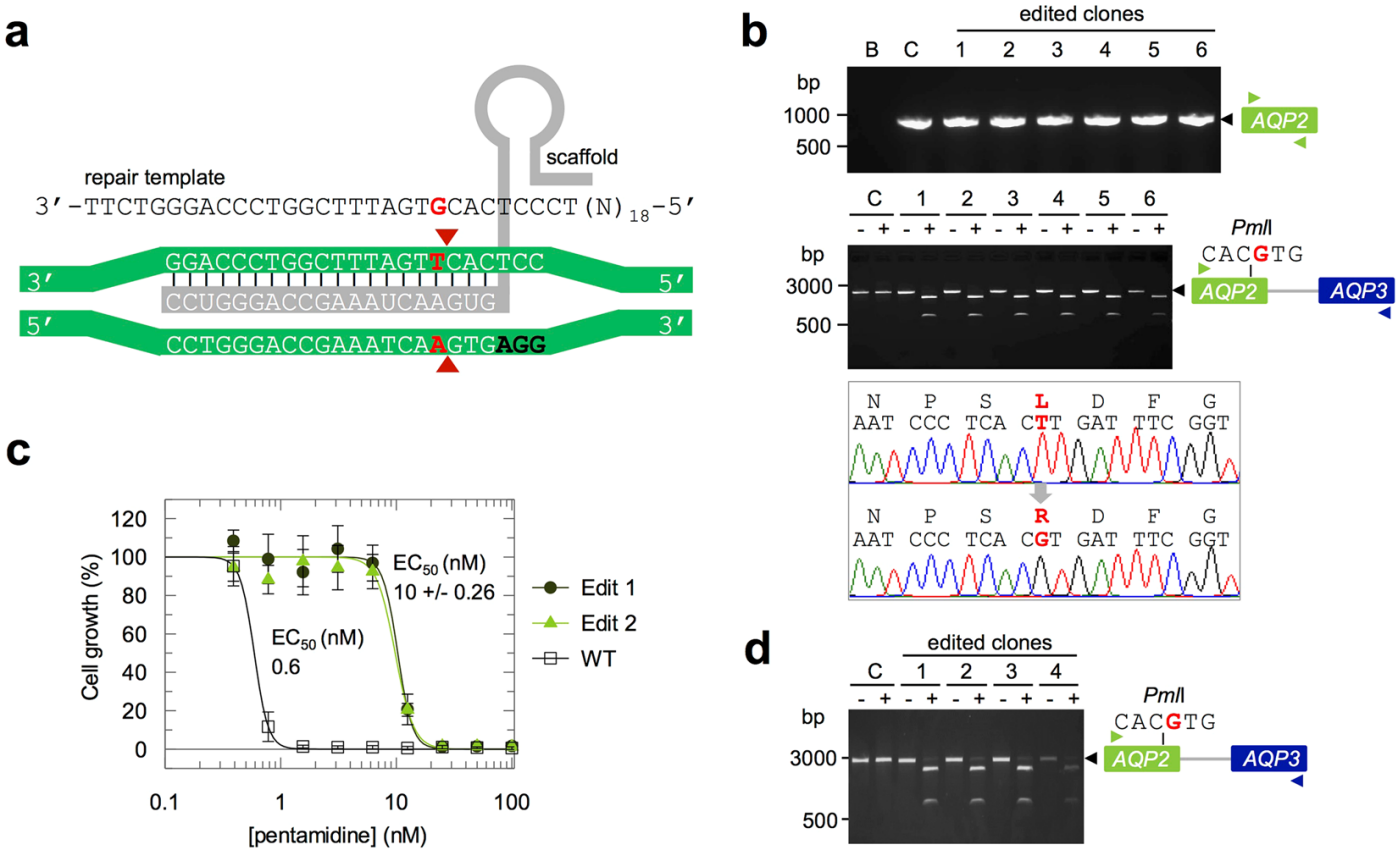
Precision base editing with Cas9. (**a**) The schematic illustrates the *AQP2*-editing strategy (one allele shown). The genomic DNA sequence is on a green background, the PAM is in bold black text, the gRNA is on a grey background and the arrowheads indicate the expected Cas9-induced break sites. The single-stranded repair template is centred on the editing site and shows the T-G edit in red letters. (**b**) Analysis of sub-clones from an edited and pentamidine-selected population. The PCR assays are as described in Fig. 2c. B, blank – no genomic DNA; C, control – wild-type genomic DNA. The *AQP2-3* PCR products were also digested with *Pml*I (lower panel) and sequencing confirmed that all six clones had the templated T^791^G/L^264^R edit (one example shown, lower trace). (**c**) Dose-response curves for pentamidine. Both edited clones analysed display a drug-resistant phenotype. WT, wild-type cells. (**d**) Analysis of the four pentamidine-resistant sub-clones among 48 sub-clones from an edited population. The PCR assay and *Pml*I digest are as described in b.

The analyses above demonstrate robust and efficient inducible editing. The edits described are site-specific and are sufficiently efficient that neither antibiotic selectable markers, nor exhaustive screening, are required to isolate edited clones. However, these edits typically result in deletions that may also affect neighbouring sequences or genes. We next sought to use this system for precision editing by co-opting the native homologous recombination machinery. This requires the introduction of a DSB adjacent to the edit site and the introduction of a DNA template containing the desired site-specific edit (Fig. 4a). We once again chose *AQP2* as the target gene for this experiment. AQP2 is an atypical aquaglyceroporin that lacks many of the conserved pore-lining residues, including the ‘aromatic arginine’ motif^30^. A leucine residue is present at this position and a single L^264^R change generates a mutant AQP2 that fails to reverse pentamidine resistance when over-expressed in *aqp2*-null trypanosomes^38^. Cas9 editing technology now presents opportunities to test the behaviour of such mutant genes in their native context. Short homology flanks are sufficient for recombination in *T*. *brucei*^13^ and single-stranded oligonucleotides can be used to edit trypanosomatid^14,22^ and metazoan genomes^39^. We, therefore, used a new gRNA, cloned in the pT7^sgRNA^ construct, that targets a sequence adjacent to the L^264^ codon (Fig. 4a). In this case, although the overall GC-content is 55%, the PAM-adjacent GC content of the new gRNA is 3/6 and may be suboptimal (Fig. 4a, see above).

We used this new ‘AQP2.2′ gRNA to assemble Cas9/sgRNA^AQP2.2^ clones. We next delivered the short (48 b) single-stranded oligonucleotide repair-template (Fig. 4a) immediately followed by induction of Cas9-based cleavage. The repair template carries the single edited base-change (Fig. 4a, red) and was delivered by electroporation while Cas9 was induced for only 24 h in this case, to maximise template-directed repair. Note that, despite the base-change, the edited alleles may remain substrates for re-cleavage, albeit at reduced efficiency.

We expected a number of possible outcomes within the resulting population of cells; no editing, non-templated deletions (as described above) or the desired edit. Un-edited cells remain pentamidine-sensitive and these were selected against by growth in pentamidine for three days, immediately following the 24 h induction period. The resulting pentamidine-resistant population was sub-cloned and six clones were subjected to an *AQP2* PCR assay. In this case, all six clones analysed yielded an *AQP2* PCR-product (Fig. 4b, upper panel, Supplementary Fig. 1), in contrast to the clones above that underwent non-templated repair (Fig. 2c). Using a second PCR assay, all six clones also yielded an *AQP2-3* PCR-product that contained a new *Pml*I restriction site introduced by the single-base edit (Fig. 4b, middle panel, Supplementary Fig. 1). Sequencing of these products confirmed the desired T^791^G/L^264^R precision edit in every clone (Fig. 4b, lower panel; Supplementary Fig. 2). This suggests that the electroporated short single-stranded oligonucleotide is used remarkably efficiently as a template and that this particular homology-directed repair pathway is favoured over the error-prone single-strand annealing pathway. To assess the impact of expression of the AQP2 - L^264^R mutant in the native context, we determined the EC_50_ for pentamidine. This revealed robust resistance and an EC_50_ that was increased approximately 17-fold relative to the wild-type control (Fig. 4c).

The remarkable efficiency of precision base editing above suggested that we may be able to isolate precision edited clones without selection to enrich for the edited cells. We, therefore, repeated the experiment above but, instead of selecting against unedited cells by growth in pentamidine, we simply sub-cloned the population immediately following the 24 h induction period. Forty-eight clones were expanded for several days without further Cas9-induction and were then tested for pentamidine-resistance. We found four clones (~8%) to be resistant and, again using a PCR-assay and diagnostic digest (Fig. 4b), found that all four were precision-edited (Fig. 4d, Supplementary Fig. 1). These results suggest that, using the Cas9-editing system described here, it will be possible to isolate clones with precision-edits at different loci, following screening of a readily manageable number of clones.

## Discussion

The versatility and accessibility of tools for genome editing have improved rapidly in recent years and Cas9-based approaches are central to this transition. Substantial progress has been made in developing these systems in trypanosomatids but an inducible Cas9 editing system has not yet been reported. Additionally, for *T*. *brucei*, double-allele editing has not been achieved without selectable markers nor have efficiencies been determined. Given these limitations, precision base editing is not readily achievable. To advance Cas9-based editing tools and approaches for *T*. *brucei*, we have established a highly efficient inducible system and have used the system for templated, homology-directed, precision base editing.

The system we describe incorporates several features that improve convenience and editing efficiency. In terms of convenience, the current strategy involves annealing a pair of oligonucleotides and ligating the product to the *Bbs*I-digested pT7^sgRNA^ construct prior to transfection. This cloning step is highly efficient, but sgRNA delivery can also be achieved in a cloning-free format. Such a cloning-free format, involving transient transfection of the sgRNA or an sgRNA-encoding PCR-product, may be suitable for strategies that do not require maximal-efficiency editing, such as when integrated antibiotic resistance markers are used to select for correctly edited cells^13^. In terms of the editing efficiency of our system, several features likely contribute; first, Cas9 was codon-optimised to increase expression in *T*. *brucei*^28^. Second, Cas9 and the sgRNA are transcribed from strong *rDNA* and T7 promoters, respectively. Third, the T7 polymerase, *Bbs*I-cloning approach and ribozyme all help to produce sgRNAs with minimal superfluous sequence, likely enhancing assembly with Cas9. In addition, tightly regulated temporal control of Cas9 expression increases the versatility of the system, also minimising off-target edits and allowing for phenotypic analysis of edited cells without potential interference from continuously expressed Cas9. Finally, and as in metazoan cells^37^, our findings support the view that gRNA sequences achieve optimal efficiency cleavage in *T*. *brucei* when the PAM-adjacent GC-content is >50%.

A major source of off-target edits may involve the non-specific association of Cas9 with native RNA. Indeed, expression of Cas9 in the absence of sgRNA has been reported to be toxic in *T*. *cruzi*^18^ and *Toxoplasma gondii*, and a “decoy” sgRNA has been used in *Toxoplasma* to reduce these effects^24^. The current system should minimise such problems in *T*. *brucei* since we constitutively transcribe the sgRNA and induce a tightly regulated Cas9 gene, which stabilises the sgRNA. Thus, cells are not exposed to Cas9 in the absence of sgRNA. Titration of Cas9-dosage can dramatically increase cleavage specificity and reduce off-target effects in metazoan cells^40^ and the inducible system now presents an opportunity to titrate Cas9 expression in *T*. *brucei* simply by reducing the tetracycline concentration in the growth medium^4^ or by inducing Cas9 for only a short period of time. Temporally constrained exposure to Cas9 is also important for precision base editing since precision-edited sequences may still be potential substrates for re-cleavage^37,40–42^. It will be worthwhile to test other versions of Cas9 in *T*. *brucei*. For example, HypaCas9 (*Sp*Cas9: N692A/M694A/Q695A/H698A) displays reduced off-target editing^43^ while xCas9 variants display broader PAM compatibility^44^.

Further optimisation of specific editing and minimising off-target edits can also be achieved by modifying the gRNA. In metazoan cells, gRNA sequences can be truncated by 2–3 nt to reduce off-target cleavage without substantially reducing on-target efficiency^45^ and these gRNAs can be further optimised to improve specific activity^37,40,41^. On the other hand, trypanosomatid genomes are relatively small at ~30 Mbp, likely presenting relatively few potential off-target sites. In addition, homologous recombination is the dominant repair pathway in *T*. *brucei*^5^ such that low-frequency off-target edits in one allele will typically be reversed, since allelic templated, homology-directed repair will be favoured over error-prone repair by end-joining. In circumstances where off-target edits must be avoided, genome sequencing and profiling of potential off-target sites will remain an option.

Established tools for the genetic dissection of *T*. *brucei* already present a range of options. Cas9-based approaches present alternative strategies and, perhaps more importantly, present completely new opportunities, for increased throughput and for marker-free and precision editing, in particular. In terms of alternative strategies, RNAi is currently the favoured approach for conditional loss-of-function studies in *T*. *brucei*, particularly for many likely essential genes. Similarly, native, single-allele tagging for protein localisation studies is readily achievable using selectable marker based constructs in *T*. *brucei*^46^ and can now be considered alongside (double-allele) Cas9-based editing options. Clearly, editing efficiency need not be high if selectable markers are to be used to enrich for cells with a desired, non-lethal edit. When an edit is lethal on the other hand, the desired editing will fail to yield long-term viable recombinant cells. In these circumstances, editing that is both inducible and close to 100% efficiency, will be a major advantage since it will facilitate the study of phenotypes associated with lethal edits that precede cell death. High efficiency will also be important for other marker-free or high-throughput approaches. In these cases, high validation rates for a desired edit will substantially reduce the number of clones that must be screened and will improve coverage across large numbers of editing sites, respectively.

Key advantages of precision editing without selectable markers are that the (double-allele) edit is generated in one step in a native context, retaining native flanking sequences that may control expression level or timing during the cell cycle or the life cycle. A recombinant AQP2 *-* L^264^R mutant was previously ectopically overexpressed in an *aqp2* null background, for example^38^. The pentamidine resistance phenotype associated with AQP2 *-* L^264^R expression is confirmed here but the prior ectopic expression approach required three genetic manipulation steps and was also associated with risks of potential artefacts associated with non-physiological expression. Thus, the current editing approach adds an important new ‘one-step, precision’ option to the genetic manipulation ‘toolbox’ for *T*. *brucei*. The efficiency reported here indicates that it is now feasible to obtain cells with edited loci without selection, by screening manageable numbers of clones.

The Cas9-based editing system we describe for *T*. *brucei* incorporates key features, including robust, temporal control of Cas9 and sgRNA expression. We show that null-clones (both alleles disrupted) can be generated at exceptionally high frequency without selection. Future applications will likely extend beyond precision edits, deletions and insertions to high-throughput approaches and the repurposing of nuclease-dead Cas9 for targeting other functionalities to specific genomic loci^12^.

## Materials and Methods

### *T*. *brucei* strains and growth conditions

Bloodstream form *T*. *brucei*, Lister 427 (MITat 1.2), clone 221a cells, 2T1 and 2T1^T7^ cells^25^ and their derivatives were grown in HMI-11 medium. Cumulative growth rates were monitored by splitting to 1 × 10^4^ cells/ml and by counting daily. Tetracycline (Sigma) was applied at 1 µg/mL. Pentamidine isethionate was from Sigma and eflornithine was from Sanofi Aventis.

### *T*. *brucei* genetic manipulation and plasmid construction

*T*. *brucei* were genetically manipulated using electroporation as described^25^. To generate the pRPa^Cas9^ construct, the *Sp*Cas9 sequence was codon optimised and synthesised (Genscript) with a La nuclear localisation signal^27^ at the *N*-terminus. The synthetic Cas9 cassette, digested with *Hin*dIII/*Bgl*II, was ligated to pRPa^c6MYCn ^^26^ [Addgene ID:69242] digested with *Hin*dIII/*Bam*HI. The resulting pRPa^Cas9^ construct was linearised with *Asc*I prior to electroporation; 2T1^T7-Cas9^ parasites were selected with hygromycin at 2.5 µg/mL and checked for sensitivity to puromycin to indicate correct construct integration. To generate the pT7^sgRNA^ construct, the *BLA* resistance cassette in pRP^c6MYCn ^^26^ [Addgene ID:69243] was replaced by a *BLE* cassette following *Ngo*MIV/*Bst*Z17I digestion. A synthetic sequence comprising a T7 promoter, tandem *Bbs*I sites, the sgRNA scaffold and an HDV ribozyme was then used to replace the expression cassette from pRP^c6MYCn^, following *Nhe*I/*Apa*I digestion. Potential gRNA sequences were identified either manually or using the LeishGEdit.net online tool^13^, and then selected based on PAM-adjacent GC-content^37^. The gRNA oligonucleotide pairs were FwgAQP2 (**AGGG**GGTGTTTGCCATGTACCCCG) and RvgAQP2 (**AAAC**CGGGGTACATGGCAAACACC); FwgAAT6i (**AGGG**GTTTAAGTTCACATTGTCGC) and RvgAAT6i (**AAAC**GCGACAATGTGAACTTAAAC); FwgAAT6ii (**AGGG**CAACGTTCGCCTTGAATATA) and RvgAAT6ii (**AAAC**TATATTCAAGGCGAACGTTG) and, for the ‘AQP2.2′ precision edit, FwgAQP2c (**AGGG**CCTGGGACCGAAATCAAGTG) and RvgAQP2c (**AAAC**CACTTGATTTCGGTCCCAGG); the overhanging ends that facilitate cloning are in bold. Oligonucleotide pairs were annealed by heating to 70 °C for 3 min followed by slow cooling. Annealed pairs were ligated to *Bbs*I-digested pT7^sgRNA^ and constructs were confirmed by sequencing. pT7^sgRNA^ constructs containing specific gRNA sequences were linearised with *Not*I prior to electroporation and parasites were selected with phleomycin at 2 µg/mL. 40 µg of single-stranded oligonucleotide (GTCTCCCCTTGCGATGAATCCCTCAC**G**TGATTTCGGTCCCAGGGTCTT) was used as a repair template, which was resuspended in 10 μL of H_2_O and delivered by electroporation as described above; the edited base is in bold.

### DNA analysis

DNA was extracted from *T*. *brucei* using a DNeasy blood and tissue Kit (Qiagen). The primer pairs used for the PCR assays were FwAQP2only (GAGCGGTGGGATGCAGATGTAC) and RvAQP2only (CCCCGAGAAGGATCGCACCG), FwAQP3only (GTGTGGTCGCCACGGTTATC) and RvAQP3only (GCGTAACCCGTTGAGTAACCG), FwAAT6only (ATGAGAGAGCCGATACAAACTTCAAC) and RvAAT6only (TCAGAGTTCAGCAATGACGCTG), Fw5430ATG (ATGGGCAACAACGGAAGTAGC) and Rv5430STOP (CTATGCCGCCAGTCGAACAC). The FwAQP2only or Fw14180 (CCAAAATCAGCGGGTTCACTGA) primers were combined with RvAQP3only for the *AQP2-3* PCR assays (lower panels in Fig. 2c).

### RNA analysis

For northern blotting, RNA was extracted from *T*. *brucei* using an RNeasy Mini Kit (Qiagen). 1.5 µg of RNA was resolved on formaldehyde agarose gels in MOPS buffer and transferred to nylon membranes by capillary blotting. A digoxigenin (DIG)-labelled riboprobe was hybridized to the membrane and the transcript was detected using an anti-DIG antibody (Roche) with a chemiluminescent CDP-star substrate (Roche). rRNA stained with ethidium-bromide was used as loading control.

### Protein analysis

Total *T*. *brucei* extracts equivalent to 1 × 10^6^ cells were separated on SDS-polyacrylamide gels and subjected to western blotting analysis according to the manufacturers’ instructions (BioRad). Blots were blocked in 5% milk in PBS-T and washes were performed in PBS-T (0.05% Tween). Blots were then cut in two, and the upper part (above 70KDa according to the pre-stained protein ladder) was probed with 1/1000 α-Cas9 primary antibody (Abcam) and 1/5000 α-mouse secondary antibody (Bio-Rad). As a loading control, the lower part of each blot was incubated with 1/20000 α-EF1α (Merck Millipore) and 1/5000 α-mouse secondary antibody (Bio-Rad).

### Drug sensitivity analysis

EC_50_ analysis was performed using the AlamarBlue method as described^47^; drug exposure was for 72 h and incubation with resazurin (Sigma-Aldrich) was for 5–6 h. Plates were read on an Infinite 200 Pro plate-reader (Tecan).

### Data Availability

The datasets generated during the current study are available from the corresponding author on reasonable request.

## Electronic supplementary material

Supplementary Fig's 1-2
