# Supplementary material for: Inducible high-efficiency CRISPR-Cas9-targeted gene editing and precision base editing in African trypanosomes
Source: Sci Rep. 2018 May 21;8:7960. doi: 10.1038/s41598-018-26303-w (PMC5962531; doi:10.1038/s41598-018-26303-w)
Supplement: Supplementary file 1 — Supplementary Fig's 1-2 [file 41598_2018_26303_MOESM1_ESM.pdf]

Inducible high-efficiency CRISPR-Cas9-targeted gene editing and precision base editing in African trypanosomes

Eva Rico, Laura Jeacock, Julie Kovářová and David Horn

Supplementary Information.

Supplementary Figures 1-2.

Uncropped gel and blots for Fig. 1.

Fig. 1b.

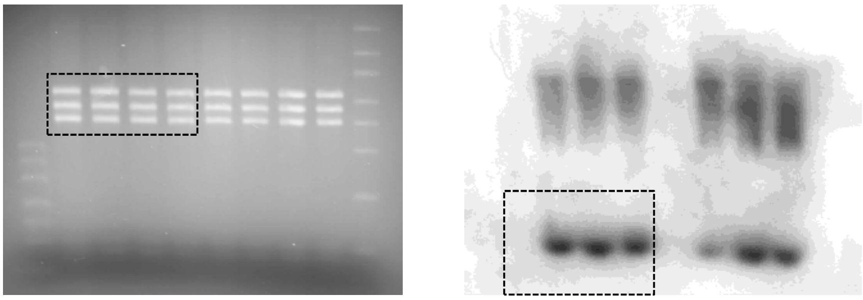

Fig. 1e.

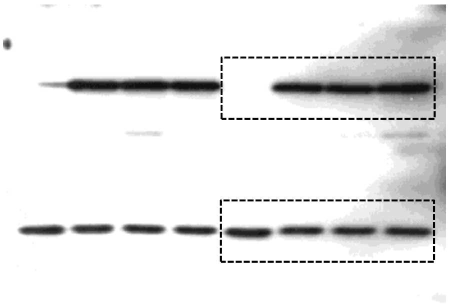

Uncropped gels for Fig. 2c.

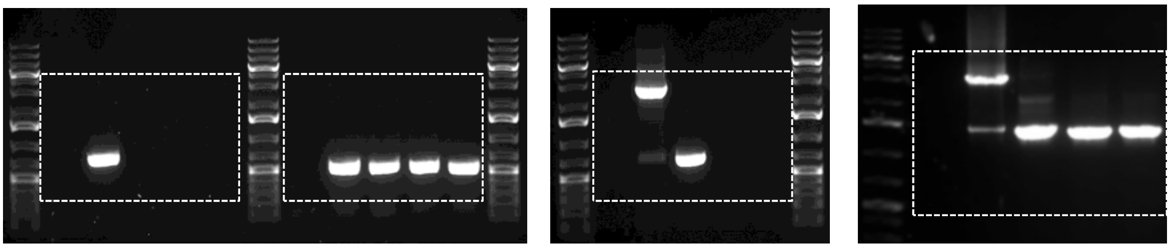

Uncropped gels for Fig. 3c.

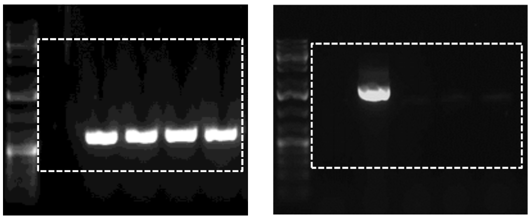

Uncropped gels for Fig. 4.

Fig. 4c.

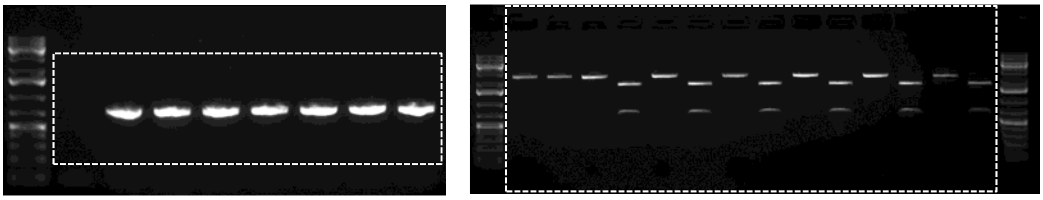

Fig. 4d.

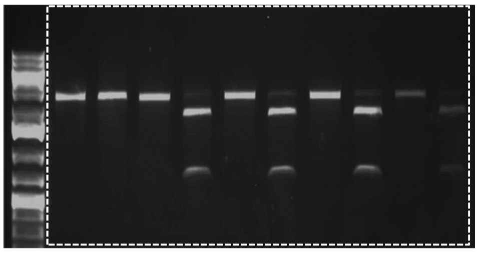

Supplementary Fig. 1. All uncropped gels and blots are shown for Figures 1-4. The areas shown in Figures 1-4 are indicated by dashed boxes. Data for a second clone are also shown in the Fig. 1 panels.

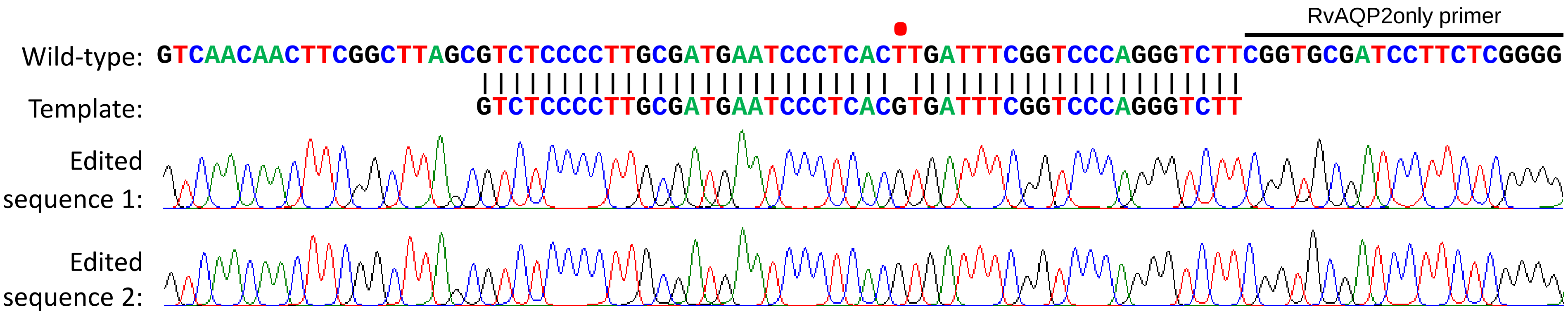

Supplementary Figure 2. Extended sequence traces from Fig 4b and for two independent clones, showing only the precision $T^{791}G$ edit and no additional changes in the template or flanking regions. The location of one of the primers used to amplify fragments for sequencing is indicated.
